# Orthopaedic Surgery in a Patient with Metal Sensitivity

**DOI:** 10.4103/0974-2077.79202

**Published:** 2011

**Authors:** Raviraj Adala, Murali Chakravarthy, Vijayakumar Srinivas, Sanjay Pai

**Affiliations:** *Departments of Orthopedic Surgery and Anesthesia, Fortis hospitals, Bannerughatta Road, Bangalore. E-mail: orthoraviraj@yahoo.com*

Sir,

Cutaneous hypersensitivity to metal is not uncommon, affecting about 10–15% of the population.[1–4] Dermal contact with metals and ingestion of metals have been reported to cause immune reactions, which may manifest as erythema, itchy papulovesicular eruptions, hives, etc.[5–10] Metals known to be sensitizers (haptenic moieties in antigens) include beryllium, nickel,[3,5,6,11] cobalt[11] and chromium. Hypersensitivity to tantalum,[12] titanium[13,14] and vanadium[12] has been reported only rarely. When patients with hypersensitivity to metals present for surgery where metallic prostheses are required, problems arise about the choice of the prosthesis. We present a case of a patient with proven metal sensitivity to cobalt, chromium, nickel and molybdenum, who required bilateral total knee replacement for osteoarthritis and was successfully managed by a titanium prosthesis.

A Caucasian lady aged 65 years, with severe osteoarthritis in both the knees requiring bilateral total knee replacement presented at our institution for receiving the bilateral knee prosthesis. The patient had previous history of allergic reaction to metal buttons in her garments, wrist watch, and metallic jewellery. She also had persistent wound discharge after ankle surgery following a fracture of the ankle a decade ago. The fracture site had earlier been fixed using plates made up of stainless steel (cobalt, chromium and nickel). Following this surgery, the persistent operative wound discharge led to the removal of the prosthesis. Subsequently, testing for allergy to metals was performed by the MELISA^®^ test, which is a blood test to measure Type-IV delayed hypersensitivity reaction (by lymphocyte transformation) to the conventionally used metals such as nickel, chromium, cobalt and molybdenum.

**Figure 1:**
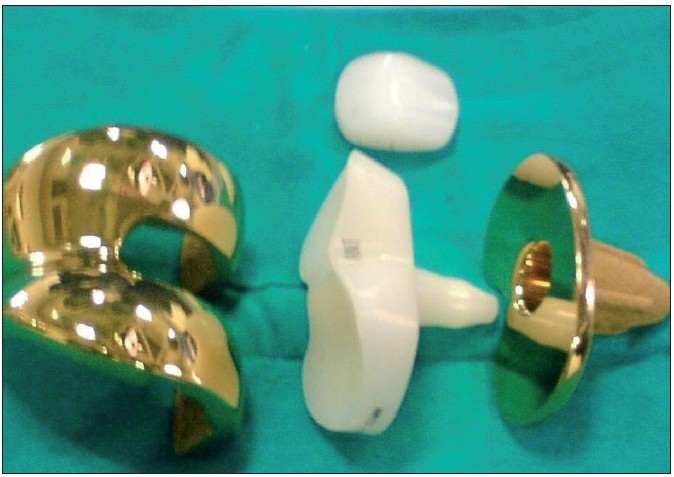
Titanium total knee prosthesis used in the patient

The MELISA test in the patient revealed strongly positive allergy to nickel and chromium and weakly positive results for cobalt and molybdenum. Based on the MELISA report, the implant to be used in our patient was chosen to be of titanium (femoral and tibia component) and a polyethylene insert was used to avoid the possible metal allergy. Since the commonly used prostheses are made up of cobalt, a completely titanium LCS total knee prosthesis (Depuy^®^) had to be specially procured [Figure 1] for the patient. Bilateral total knee replacement was performed in two surgeries staggered over 4 days. The patient was discharged a week later without any problems.

The case demonstrates the importance of metal allergy in patients undergoing metal prosthetic surgery. All metals in contact with biological systems undergo corrosion. This electrochemical process leads to the formation of metal ions, which may activate the immune system.[15] If cutaneous signs of an allergic response appear after the implantation of a metal device, metal sensitivity should be considered. In the absence of signs and symptoms mentioned above, as in our patient, nonhealing of a postoperative wound in the absence of infection should raise doubts of metal allergy. The testing for delayed-type hypersensitivity for such patients can be conducted either *in vivo* by skin testing (patch testing or intradermal testing) or *in vitro* by lymphocyte transformation testing and leukocyte migration inhibition testing using the patient’s blood. Titanium appears to offer the least allergic profile in comparison to other metals and was therefore chosen in our patient. The impurities in titanium might induce allergies at times, although rare.

Our case demonstrated the need for proper investigation with tests such as MELISA and also the need for proper history in all such patients.
